# Supplementary material for: Human genomics of the humoral immune response against polyomaviruses
Source: Virus Evol. 2021 Jun 11;7(2):veab058. doi: 10.1093/ve/veab058 (PMC8438875; doi:10.1093/ve/veab058)
Supplement: veab058_Supp [file veab058_supp.zip › Supplementary File for Review.docx]

# Supplementary Figures


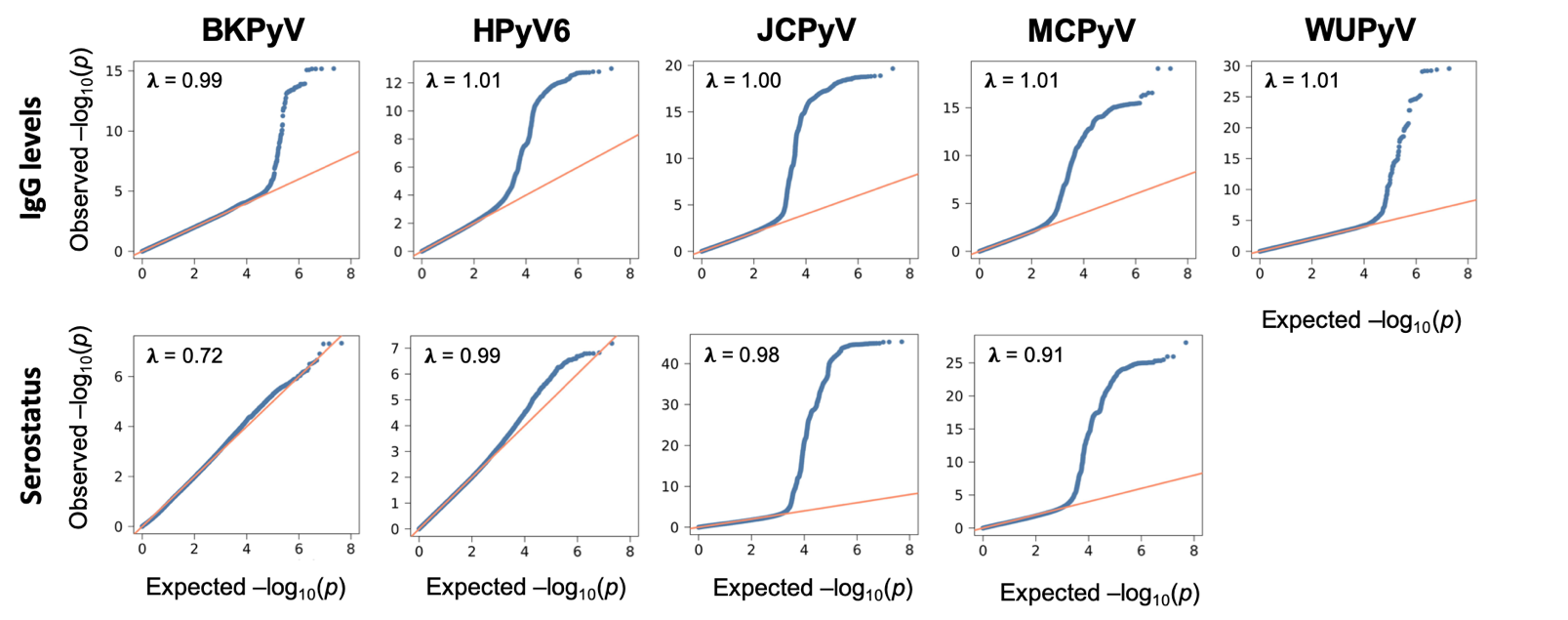


**Figure S1. Quantile-Quantile (Q-Q) plots of SNPs for association to IgG levels and serostatus in the meta-analysis of CoLaus, UKB and GRAS.** On the y-axis, the observed p-values (blue dots) are plotted against the expected p-values under the null distribution. The red line indicates the distribution of SNPs under the null distribution. Lambda (λ) denotes the genomic control inflation factor.


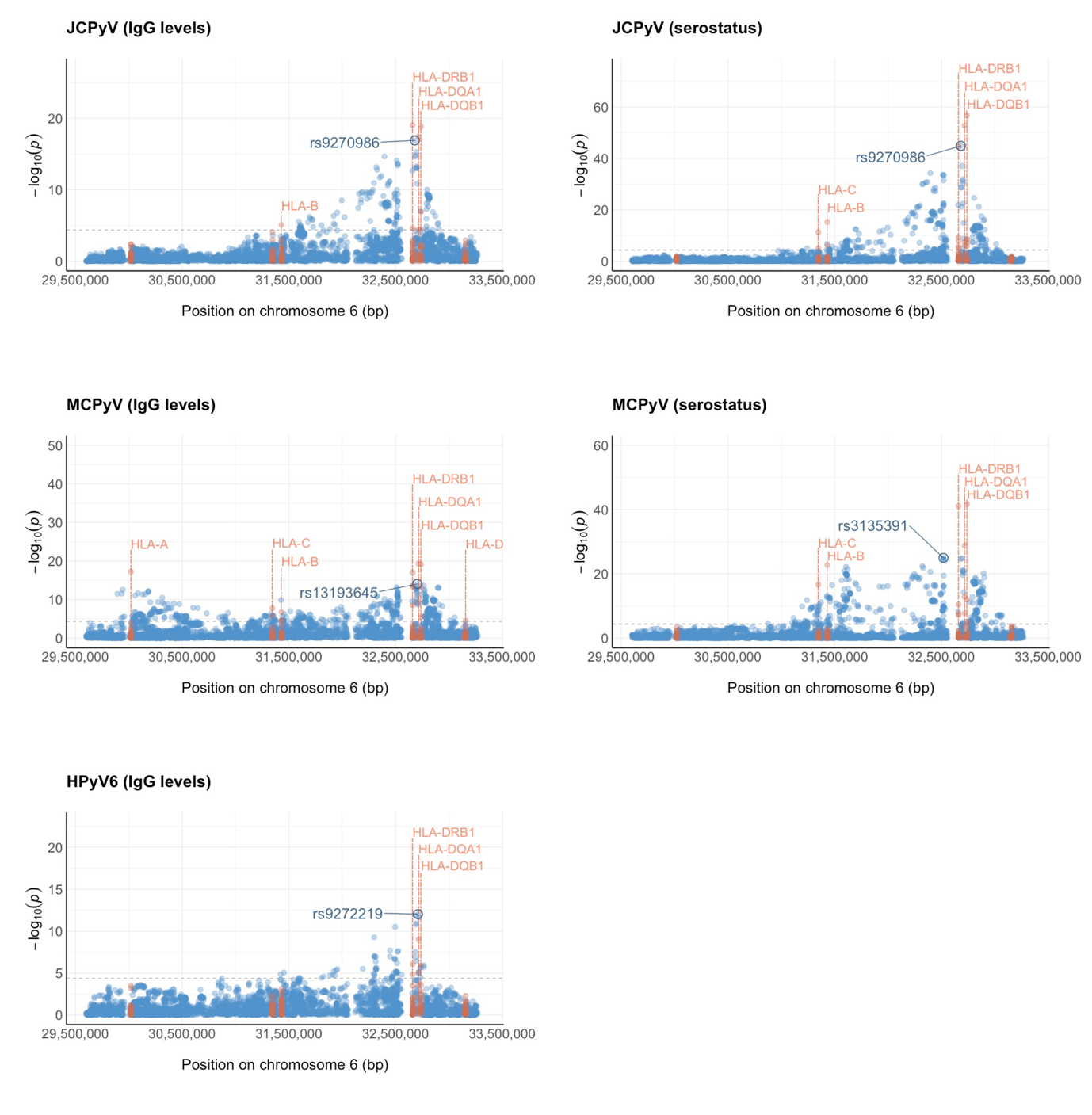


**Figure S2.** **Regional association plot of SNP2HLA results for BKPyV, JCPyV and MCPyV.** For each SNP (blue) and each classical HLA allele (orange), the P-value (in -log_10_) is plotted against its position in the MHC genomic region on chromosome 6 (UCSC hg17, NCBI Build 35). The most significant association was observed for rs9270986. The dashed horizontal line indicates the threshold for HLA-wide significance (P=4.3e-05). The annotated dashed orange vertical lines indicate the positions of the significant HLA alleles. The circled point represents the top associated SNP.

#
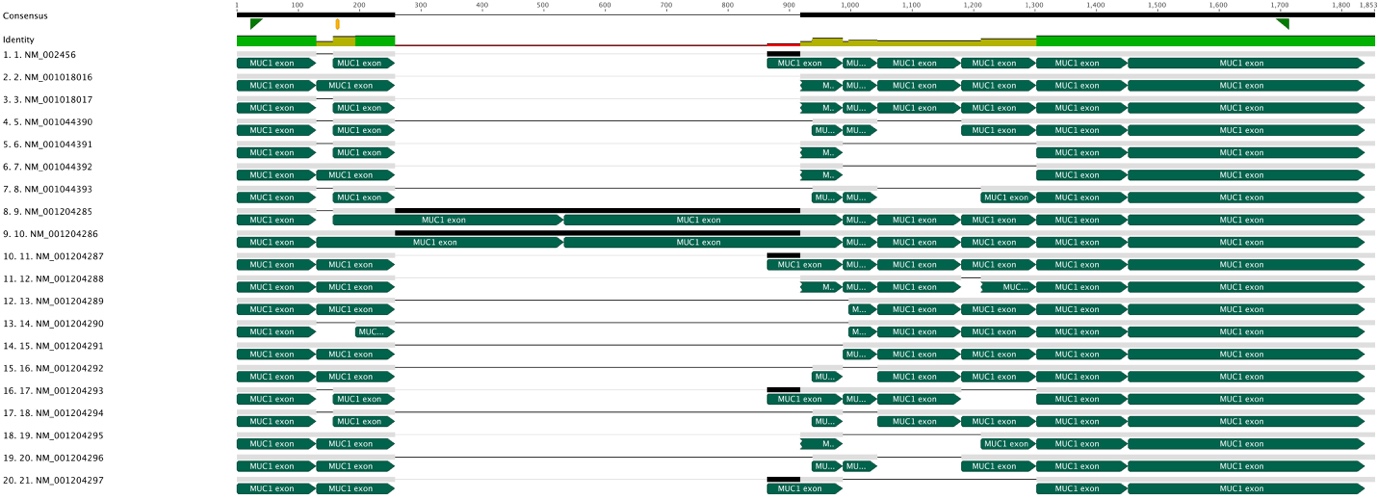


**Figure S3.** **Structural representation of *MUC1* isoforms.** The exons are represented with green boxes.

#
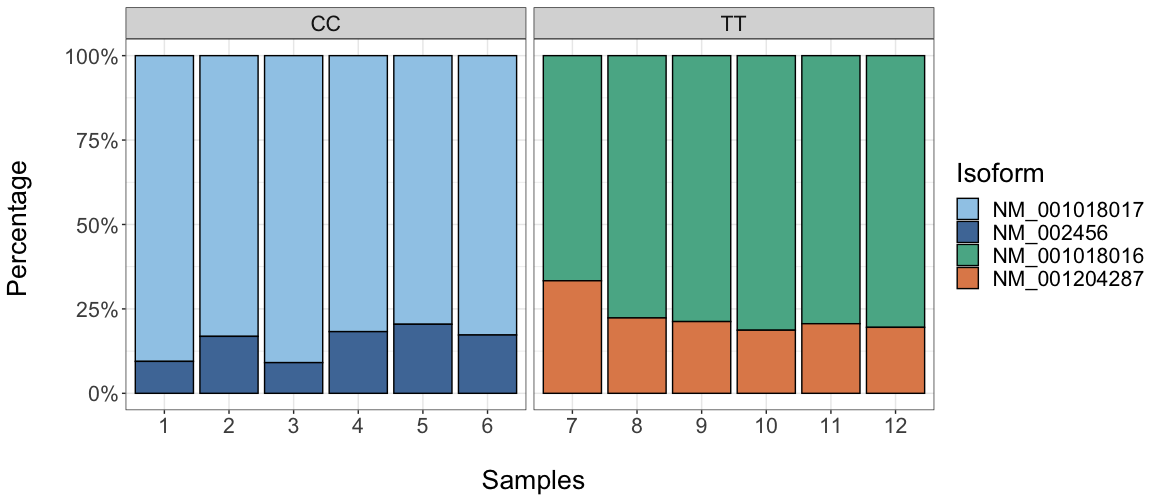


**Figure S4. Percentage of reads, as mapped to the different *MUC1* isoforms.** The plot shows the percentage of mapped reads for the 12 stomach RNA samples from GTEx, 6 of which from homozygous carriers of the rs4072037 major allele T (left panel), and 6 from homozygous carriers of the minor allele C (right panel).

Supplementary Tables

**Table S1.** Results from the serological analyses of rs4072037 for six different *H. pylori* and four EBV antigens in CoLaus study.

| **Pathogen** | **Antigen** | **Prevalence (%)** | **Beta** | **SE** | **P-value** |
| --- | --- | --- | --- | --- | --- |
| **EBV** | VCA p18 | 93 | 7.1e-03 | 2.3e-02 | 7.6e-01 |
|  | EBNA | 90 | 9.6e-03 | 2.3e-02 | 6.8e-01 |
|  | Zebra | 88 | -1.9e-03 | 2.3e-02 | 9.3e-01 |
|  | EA-D | 77 | 4.2e-02 | 2.3e-02 | 7.0e-02 |
| ***H. pylori*** | HP1564 OMP | 28 | -2.0e-02 | 2.3e-02 | 3.8e-01 |
|  | HP10 GroEL | 27 | -8.5e-03 | 2.2e-02 | 7.1e-01 |
|  | HP547 CagA | 17 | -9.7e-03 | 2.3e-02 | 6.7e-01 |
|  | HP887 VacA | 17 | 2.5e-02 | 2.3e-02 | 2.7e-01 |
|  | HP73 UreaseA | 15 | -1.8e-02 | 2.3e-02 | 4.3e-01 |
|  | HP875 Catalase | 14 | -1.4e-03 | 2.3e-02 | 9.5e-01 |
| **WUPyV** | VP1 | 96 | -2.5e-01 | 2.3e-02 | 6.2e-28 |

**Table S2.** Read counts, as mapped to the different MUC1 isoforms.

| **Genotype** | **Sample** | **NM_001018017** | **NM_002456** | **NM_001018016** | **NM_001204287** | **Total** |
| --- | --- | --- | --- | --- | --- | --- |
| **CC** | **1** | 67 | 7 | 0 | 0 | 74 |
| **CC** | **2** | 69 | 14 | 0 | 0 | 83 |
| **CC** | **3** | 30 | 3 | 0 | 0 | 33 |
| **CC** | **4** | 85 | 19 | 0 | 0 | 104 |
| **CC** | **5** | 105 | 27 | 0 | 0 | 132 |
| **CC** | **6** | 91 | 19 | 0 | 0 | 110 |
| **TT** | **7** | 0 | 0 | 74 | 37 | 111 |
| **TT** | **8** | 0 | 0 | 80 | 23 | 103 |
| **TT** | **9** | 0 | 0 | 63 | 17 | 80 |
| **TT** | **10** | 0 | 0 | 87 | 20 | 107 |
| **TT** | **12** | 0 | 0 | 77 | 20 | 97 |
| **TT** | **12** | 0 | 0 | 70 | 17 | 87 |
| **Total** | | 447 | 89 | 451 | 134 | 1121 |
